# Optimization of the SAG Grinding Process Using Statistical Analysis and Machine Learning: A Case Study of the Chilean Copper Mining Industry

**DOI:** 10.3390/ma16083220

**Published:** 2023-04-19

**Authors:** Manuel Saldaña, Edelmira Gálvez, Alessandro Navarra, Norman Toro, Luis A. Cisternas

**Affiliations:** 1 Faculty of Engineering and Architecture, Universidad Arturo Prat, Iquique 1110939, Chile; notoro@unap.cl; 2 Departamento de Ingeniería Química y Procesos de Minerales, Universidad de Antofagasta, Antofagasta 1270300, Chile; luis.cisternas@uantof.cl; 3 Department of Metallurgical and Mining Engineering, Universidad Católica del Norte, Av. Angamos 0610, Antofagasta 1270709, Chile; egalvez@ucn.cl; 4 Department of Mining and Materials Engineering, McGill University, 3610 University Street, Montreal, QC H3A 0C5, Canada; alessandro.navarra@mcgill.ca

**Keywords:** SAG mill, comminution processes, artificial intelligence algorithms, modeling, optimization, mineral processing

## Abstract

Considering the continuous increase in production costs and resource optimization, more than a strategic objective has become imperative in the copper mining industry. In the search to improve the efficiency in the use of resources, the present work develops models of a semi-autogenous grinding (SAG) mill using statistical analysis and machine learning (ML) techniques (regression, decision trees, and artificial neural networks). The hypotheses studied aim to improve the process’s productive indicators, such as production and energy consumption. The simulation of the digital model captures an increase in production of 4.42% as a function of mineral fragmentation, while there is potential to increase production by decreasing the mill rotational speed, which has a decrease in energy consumption of 7.62% for all linear age configurations. Considering the performance of machine learning in the adjustment of complex models such as SAG grinding, the application of these tools in the mineral processing industry has the potential to increase the efficiency of these processes, either by improving production indicators or by saving energy consumption. Finally, the incorporation of these techniques in the aggregate management of processes such as the Mine to Mill paradigm, or the development of models that consider the uncertainty of the explanatory variables, could further increase the performance of productive indicators at the industrial scale.

## 1. Introduction

Minerals and metals in many cases support national economies, providing strategic raw materials for industrial activities that are sources of input for almost all sectors of the global economy. Extraction and production processing encompass various activities, from highly mechanized industrial mining to informal artisanal miners. Mineral production (especially copper) has been growing in recent years. In fact, the estimated global copper mining production increased slightly to 21 million tons in 2021 from 20.6 million tons in 2020 [1]. Additionally, a report generated by the International Copper Study Group indicates that since 1900, when copper production was less than 500 thousand tons, world copper mine production has grown by 3.1% per year, reaching approximately 21 million tons in 2021 [2].

In the Chilean copper industry, approximately 70% of processing takes place by pyrometallurgical routes or flotation processes, processes that generate large environmental liabilities, such as tailings dams (negative externalities that have been reduced in recent years [3,4,5,6]), while only 30% takes place by hydrometallurgical routes, which is explained by the depletion of deposits of oxidized copper minerals. Additionally, it is estimated that by 2027, the ratio of pyrometallurgical to hydrometallurgical processes will be 80% to 20% [7]. This change is due to the depletion of deposits of oxidized copper minerals, processed by acid leaching, which has led the copper industry to process more complex copper minerals. This has resulted in more complex threads, requiring more resources and energy consumption in the comminution stage [8,9,10], mainly in minerals whose downstream processing requires smaller particle sizes.

The comminution process is a key stage within any mining worksite since most of the energy invested in the operation is concentrated on achieving a size reduction in the material (accounting for an average of 53% [11]) through both the crushing and the grinding processes [12,13]. In the crushing stage, the rock is crushed by vibratory movements, reducing the object’s volume into a series of smaller or more compact particles to obtain a finer material, which will depend on mechanisms such as chipping, abrasion, and the underlying characteristics of these mechanisms, such as the frequency of collision or the rate of loading and/or unloading into the rotary mill [14]. Then, the grinding process comprises a further reduction in the particles, adding water to the mineralized material in sufficient quantities to form a milky fluid, and adding the reagents necessary to carry out the downstream processes, such as flotation, a process that is strongly influenced by the grinding medium and the grinding method which will vary the properties of the surface and the roughness of the minerals [15]. These processes of size reduction are collectively called comminution [16]. Thus, this process aims to liberate and concentrate the mineral particles contained in mineralized rocks and particles recovered downstream in the froth flotation stage, which involves the separation of valuable ore from other minerals [17].

The run-of-mine (ROM) materials that feed copper processing plants are varied, covering particles finer than 1 (mm) to fragments thicker than 1 (m) in diameter. Hence, the objective of crushing is to reduce the size of the largest fragments until a maximum uniform size of 0.5 inches (1.27 cm) is obtained [18]. This usually takes place by combining three pieces of in-line equipment that reduce the fragments in stages: the primary stage, secondary stage, and tertiary stage. The configuration of comminution circuits mainly depends on the mechanical properties of the mineral and the requirements that comminution products should meet from the scope of downstream separation processes; therefore, the number of stages strictly depends on the operational conditions of the mining worksite. Subsequently, through grinding, the size of the mineral-bearing particles is reduced to obtain a maximum granulometry of 180 microns (0.18 mm), which finally allows the release of most of the copper minerals in the form of individual particles [19].

As grinding is a core pyrometallurgical process, the efficiency and continuous improvement of comminution processes have been the principal focus in the last two decades, where semi-autogenous grinding (SAG) is considered the grinding system par excellence (at least in the Chilean case). The advantages of SAG grinding are mainly related to a simpler layout, with fewer stages and pieces of comminution equipment in concentrator plants, which translates into less investment. Currently, AG/SAG grinding is used in most of the mining sites in Chile, and the layouts vary depending on the requirements of each concentration plant, such as plants that have the potential to make their circuits more efficient by modifying their design or operating conditions [20].

Due to the complex nature of the SAG grinding process, developing abstractions that represent the dynamics of SAG milling have the potential to contribute both to obtaining a better understanding of the process and to simulating or optimizing its performance. Considering the highly non-linear nature of the SAG milling process [21], the robustness of machine learning techniques (such as ANN [22,23], random forest [24,25], regressions [26,27,28], Bayesian networks [29,30], Malmquist and DEA [31], fuzzy logic and ANFIS [32,33], support vector machines [34,35], principal component analysis [36], and ensemble methods [37], among others), the potential of these techniques to model complex process dynamics, and the shortage of case studies in the literature in respect to SAG grinding modeling using machine learning techniques in industrial contexts. In this work, machine learning techniques for the development of hypothesis tests (prior modeling) and the application of corrective measures in order to increase process productivity are devised. The structure of this work is as follows. A state of the art overview is presented in the background section with respect to the modeling of the SAG grinding process. In the Materials and Methods section, a description of the case study, the objectives, and the scope are stated. We give a description of the machine learning algorithms used to generate the digital model of the SAG grinding process, with the respective performance measures. In the Results and Discussions section, the application of machine learning to the modeling process is presented, including the correlation of the variables, the efficiency of the algorithm adjustment, and the evaluation and discussion of the studied hypothesis. Finally, conclusions are drawn based on the performance of the digital model in terms of the operational performance indicators of the SAG mill.

## 2. Background

The SAG grinding process has been modeled by various authors, either generating explanatory models of the grinding process, with the aim of modeling, simulating, and optimizing the individual process, as well as integrating it into aggregate processes, such as the mine-to-mill (M2M) paradigm, a practice that has generated the capacity to analytically assess aspects of mining and processing (primarily the comminution process) and to run models and simulations to predict the effects of changes at the mine on downstream processing [38]. It is possible to find different trends in the modeling, design, simulation, and optimization of complex systems such as mineral processing using techniques such as computational fluid dynamics, discrete element simulation, surface response methodology, machine learning algorithms such as artificial neural networks, support vector machines or random forest, and uncertainly analysis or sensitivity analysis [39]. These techniques have been applied to different fields of practical industry [40], such as machining processes [41], chemical and process industries [42], geomechanics [43], the development of hybrid intelligent systems (combining human intelligence with artificial intelligence) [44], industrial control systems [45], decision support systems [46,47], applications in the pharmaceutical industry [48], integration through digital twins and Industry 4.0 in the food industry [49], improvement in the efficiency of industrial boilers through the detection, diagnosis, and forecasting of failures [50], and applications of discrete event simulation to metallurgical processes [51,52]. Directly related to the work carried out in the present manuscript, there was a survey of applications of machine learning algorithms in mineral processing, differing in categories such as data-based modeling, fault detection and diagnosis, and computer vision [53].

The literature review highlights works such as a study of the impact of the design of the flow diagram of a grinding circuit on the benefit of iron ore [54], the evaluation of the impact of the active control of the eccentric speed, and the adjustment of the closed circuit of the cone crusher in the performance and/or the energy efficiency of the SAG circuit, which was implemented in Matlab/Simulink as a dynamic model of the circuit of a mill crusher of SAG boulders [55]. Then, different theoretical models applied to represent the dynamics of mineral processing are exposed, both those embodied in mathematical relationships or equations and models based on machine learning.

As usual, rotary mill power equations are derived from mechanics, and the equations used for the prediction of power output by balls/AG/SAG mills [56,57,58]. However, none of these models consider the size distribution of the material in the feeding as a design variable. Many mine-to-mill (M2M) works [59,60,61] have shown the influence of ROM (in addition to mineral characteristics such as hardness, lithology, and alterations) in the performance of the comminution process, proving that the manipulation of fed material size affects the process efficiency [60]; although, it should be noted that in many cases, the size distribution of feeding is even more influential than mineral characteristics [59]. Morrel [62], on the other hand, relates the feed mineral, mill geometry, and operating conditions with the specific energy of the circuit. In the same M2M line, it has that the feed size distribution directly affecting the SAG mill load, which directly affects the power of the mill, and in turn impacts its efficiency [63].

Silva et al. [64] fit a power equation and an energy equation specific to the design of SAG mills, using them to predict energy consumption as a function of mill size, internal load level and density, critical speed, and a variable representing the size distribution of the feed (not just the F_80_). Dong et al. [65] analyzed the factors that influence energy consumption in the grinding and flotation processes in a gold treatment plant, establishing three models for the prediction of energy consumption: regression models, artificial neural networks (ANNs), and a hybrid model of a genetic algorithm (GA)-ANN, which presented the highest precision in the prediction of the response. Razani et al. [66] evaluated the effect of initial feed size range on the amount of grind product and its size distribution in order to find the particle size range to achieve the maximum grind product. Lucay et al. [67,68] demonstrate that uncertainty analysis (UA) and global sensitivity analysis (GSA) are useful tools for identifying operating conditions for grinding systems under uncertainties.

Asghari et al. [69] did not generate an integrated model of the production dynamics of an SAG mill, but they studied the effects of ore characteristics and operating parameters on mill performance using mathematical models, such as t_10_ or the Bond work index, in addition to the particle shape of the mill product to monitor breakage events as a function of ore strength. Lvov et al. [70] and Marijnissen et al. [71] developed a model of a wet SAG mill based on the discrete element method and computational fluid dynamics (CFD), which allows for determining the energy grossness ratios of the grinding process (SAG) under certain conditions [70], or to obtain the velocities and collision angles of a representative group of particles in the mill [71]. Focused on optimizing SAG grinding performance, Behnamfard et al. [72] applied the properties of feed ore, including hardness and particle size distribution, to improve the operation of an AG mill, developing a model for the prediction of mill energy consumption, a variant of the MINNOVEX model [73]. Behnamfard et al. [72] aimed to reduce energy consumption and increase mill performance by adding larger lumps and lowering the mineral’s hardness in the feeding.

On the other hand, focusing on machine learning techniques shows has that these have been increasing in presence and impact in a wide variety of research fields, highlighting their application in the study and modeling of processes in the field of mineral processing [53,74]. Many prominent review articles have discussed [75,76,77,78] and applied [79] the potential need for process engineers to take advantage of techniques such as applied mathematics and statistics, machine learning (ML), and artificial intelligence (AI).

Checking the above, in the literature review, it is possible to find a lots of applications of ML to the study of mineral processing dynamics, highlighting the following applications to the grinding phase: analysis of the mechanisms and measurement methods for the load of a mill based on mechanical vibrations and acoustic signals [80]; the impact of blast fragmentation control on increased mill production [81]; the development of a dynamic model of operation of an SAG mill using equations based on the conventional non-stationary population balance approach [82]; the identification of the best operating conditions with which to identify the cut size of optimal grinding to reduce metal losses in flotation circuits using a gradient recovery model [83]; case studies of grinding circuit modeling using time series analysis or the adjustment of vector machine algorithms of support [10] in order to analyze descriptor variables, such as power or temperature; predicting breakage and the evolution of rock size and shape distributions in AG/SAG mills [84]; models of power and specific energy consumption based on the distribution of the size of the mineral feed [64]; inferential measurement of SAG mill parameters [85,86,87,88,89]; multicomponent phenomenological modeling, which represents the performance of an SAG mill as a function of the distribution and components of the mineral feed [21]; and modeling of energy consumption prediction [90,91], among others.

Additional applications of machine learning to SAG modeling put the focus on the control of SAG grinding mill circuits, predicting power consumption [90,91], one of the main concerns of plant operators for years. In Hadizadeh et al. [92], an advanced distributed control system (DCS) is developed for the successful control of mineral processing plants, presenting the basis of an expert fuzzy supervisory controller for SAG mill circuits. In the proposed controller by Hadizadeh et al. [92], the fuzzy system calculates the optimal set points for the DCS control loops of the plant, allowing them to change the manipulation parameters to reach the new set points. In Avalos et al. [91], several predictive methods based on ML were studied for the real-time forecast of the energy consumption of an SAG mill (depending on variables such as feed tonnage, bearing pressure, and mill speed), among which are: polynomial regression, k-nearest neighbor, support vector machine, multilayer perceptron, short-term memory, and closed recurrent units (deep learning). In Kahraman et al. [90], a data-driven multi-rate (MRA) method was developed to better predict the energy consumption of a semi-autonomous grinding mill (SAG) using a deep neural network as the prediction model for the MRA method. In Avalos et al. [93], an operational definition of relative hardness is proposed and applied to SAG mill operating datasets to train a deep neural network architecture in order to forecast the next operating relative hardness, achieving accuracies greater than 80%. In Olivier et al. [94], decision trees were used to model the decision-making of the operator when deciding whether to remove material of critical size from the circuit to prevent the mill from overloading, demonstrating that the model specification can be used to identify the causes of events of interest, in addition to extracting rules to understand why and when the operators make a particular decision.

Chelgani et al. [95] used explainable artificial intelligence to build a conscious laboratory that could be a strategic approach to digitizing a high-precision grinding roll (HPGR) system, predicting particle size throughput through the conjunction of techniques such as SHAP (SHapley Additive exPlanations; SHapley values indicate how each point of a variable can contribute to the prediction, a single linear and nonlinear simultaneous multivariate evaluation) and XGBoost (extreme gradient boost model). The results obtained by Chelgani et al. [95] indicate that SHAP and XGBoost could accurately model the relationships between the operational variables of an HPGR. Finally, and not directly focused on production modeling, Azizi et al. [96] investigate the application of supervised learning algorithms (single- and multiple-kernel SVM regression analysis and artificial neural networks) to model the wear rate of grinding media as a function of multiples inputs factors, concluding that multi-kernel SVM can be used efficiently to remodel and model grinding ball wear rates.

## 3. Materials and Methods

### 3.1. Case Study

**Figure 1:**
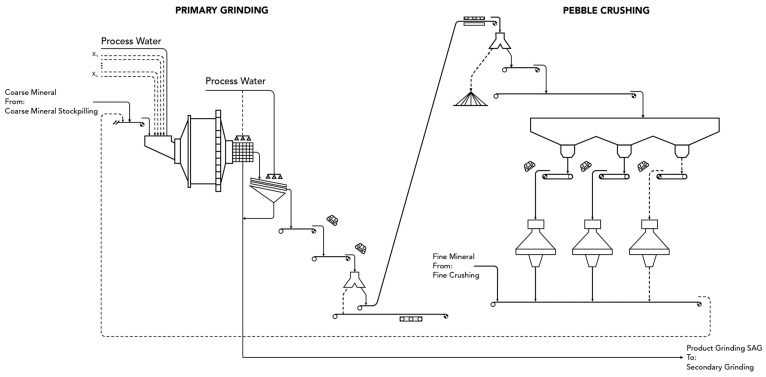
Primary grinding circuit layout.

The case study corresponds to the grinding process of a copper concentrator plant, whose operation is based on the open-pit exploitation of a mineral deposit located in the Antofagasta region in northern Chile. The main grinding line (primary grinding) of the concentrator plant is made up of an SAG mill (see Figure 1), whose operation is intended to be optimized after adjusting models based on statistical modeling and machine learning. The concentrator comminution circuit has a configuration that includes a single SAG mill with dimensions of 12.2 m × 7.3 m, followed by two ball mills in parallel (secondary grinding). Additionally, the pebble produced by the mill is returned to the SAG mill.

It is considered that there is an opportunity to increase production by focusing efforts on reducing the particle size of the SAG mill feed, supported by a mine-to-mill strategy that contributes to reducing the fragmentation of the ore fed. The optimizing production as a function of mill rotational speed is also considered, for each liner age configuration, since it is intuited that the rotational speed is higher than required, considering the mineralogical characteristics of the material fed.

The collection of historical operation data considers 12 operational variables/parameters for 27 months of operation (see the domain in Table 1), measured minute by minute, which are some of the input variables for the generation of a representative model of the SAG mill. The independent variables are as follows: P_80_; water in feeding; mill rotational speed; mill pressure; stockpile level; sump level; mineral hardness; solid percentage in feeding; pebble; granulometry thicker than 100 mm; granulometry finer than 30 mm; and liner age. The response variable is the production in tons per hour (TpH) and the energy consumption (MW).

The variables/parameters considered in the sampling are described below:

- P_80_: Size of the mesh opening that allows the passage of 80% of the granulometry.
- SAG water feeding (m^3^/h): Water flow feeding to the SAG mill.
- SAG rotational speed (RPM): Mill rotational speed.
- SAG pressure (PSI): Mill fill or load level.
- Stockpile level (m): Stockpile level in the feeding stack.
- Sump level (m): Thicker downloading pool at the SAG mill.
- Hardness: Resistance offered by the mineral to abrasion or scraping.
- Solids in the feeding (%): Percentage of solids in the feed pulp.
- Pebble (TpH): Pebble (pebble, chunks, or small stones) are the result of mineral grinding. These are hard materials and are difficult to reduce to a smaller size in the SAG mill.
- Granulometry > 100 mm (%): Percentage of the ore feed whose granulometry is greater than 100 mm.
- Granulometry < 30 mm (%): Percentage of the ore feed whose granulometry is less than 30 mm.

Liner age (months): Categorical variable. Age of the mill liners. The liners are part of the mill and act as protective jackets for the internal shell (hull), which in turn wear out over time due to the strong and constant internal impact that occurs between the ore load and the balls of steel. This wear requires that mining organizations perform predictive maintenance.

**Figure 2:**
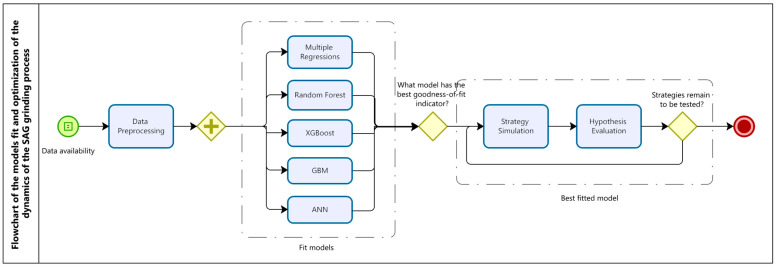
Flowchart of the model’s fit and optimization of the dynamics of the SAG grinding process (developed in Bizagi Modeler software [97]).

The proposed process for optimizing the SAG grinding dynamics (see Figure 2) considers the adjustment of machine learning algorithms (prior preprocessing of the available data) in order to determine the model that presents the best goodness-of-fit indicators. Then, once the best model has been identified, different strategies could be simulated, evaluating the hypothesis test(s) associated with each strategy and finding the optimal configurations of the variables that maximize the productive indicators.

The design of the application of machine learning techniques considers the improvement of the performance of the SAG mill using a study of the following initiatives (prior determination of the best-fitted model):

- Mine-to-mill (M2M): Identifying the current impact and potential for optimizing the crushing process before the SAG grinding process. The strategy assumes the maximization of the percentage of the granulometry in the feed that is finer than 100 mm to reduce the processing time of the SAG mill.
- SAG parameters: Optimal operation for scenarios of liner age and hardness of the feed.

The representative model of the SAG mill is used for testing the following hypotheses:

- **H_1_**: TpH SAG can be improved by M2M.
- **H_2_**: TpH SAG can be optimized by adjusting the following SAG mill parameters: pressure (load level), liner age, and rotational speed (considering hardness and fragmentation in the fed material).

### 3.2. Objectives and Scope

This work is focused on checking productivity improvement strategies, specifically in the SAG grinding process of a copper concentrator plant. It intends to establish procedures that improve productivity and reduce energy consumption, based on better management of operating variables, which can be controlled and adjusted to improve the efficiency of the process.

The use of statistical and machine learning techniques is due to the complexity of the modeled process, its non-linear dynamics, and the lack of theoretical or empirical models representative of the process. The model developed in this work includes variables that are not usually incorporated into conventional models, such as liner age. A representative model of an SAG mill can be used to optimize productive indicators, test hypotheses, and simulate probable or expected scenarios, adding value to mineral processing.

### 3.3. Statistical Analysis and Machine Learning

Machine learning involves computer programs or algorithms that automatically improve and/or adapt their performance through experience. Machine learning has many things in common with other domains, such as statistics and probability theory (understanding the phenomena that have generated the data), data mining (finding patterns in the data that are understandable to people), and cognitive science (where human learning aims to understand the mechanisms underlying the various learning behaviors exhibited by people, such as concept learning, skill acquisition, strategy change, etc.) [98]. Machine learning aims to devise learning algorithms that learn automatically without human intervention or assistance, generating methods by which the computer creates its program based on examples or samples that are provided to it [99].

Machine learning algorithms are used due to the potential to test hypotheses, analyze the high volume of data available today, make quick decisions, and test multiple scenarios and strategies. Machine learning provides new skills and abilities to the organization; these include data science, autonomy, and artificial intelligence. There are different methods of machine learning. However, among the most used methods are regression (simple, multiple, and logistic, among others) [26,27], random forest, XgBoost (a scalable tree boosting system) [100], a gradient boosting machine (GBM) [101], and artificial neural networks (ANNs) [102], among others.

Decision trees were developed using the Ranger [103] and XgBoost [100] packages in version 4.0.5 of the R programming language [104] (these packages are implementations used for the rapid calculation of random forests for high-dimensional information). Multiple regression and ANN applications were developed using the scikit-learn [105] and Keras [106] libraries, respectively, in Python 3.7.0 [107]. RStudio was the application platform of R, while the Python application was developed through Jupyter Notebooks.

Finally, the historical data were divided into two groups, i.e., the training set (70%) and the validation set (30%), while the fitted model was used for estimating the production after the application of the M2M strategy and simulating the production distribution at different values of the mill rotational speed and liner age factors.

#### 3.3.1. Regression Analysis

Linear regression analysis is a statistical technique used to study the relationship between variables [26]. Both in cases of two variables (simple regression) and more than two variables (multiple regression, MR), regression analysis can be used to explore and quantify the relationship between a dependent or criterion variable (Y) and other independent or predictor variables ($x_{1},x_{2},\ldots ,x_{n}$), as well as to develop a linear (or non-linear) equation for predictive purposes. Furthermore, a conventional regression analysis is associated with a series of diagnostic procedures, which inform the analysis of stability and suitability and provide clues on how to refine the analysis [108]. The generalization of the regression models is presented in Equation (1).

(1) $$Y=f\left(X\right)=\beta_{0}+\sum_{i=1}^{n}\beta_{i}x_{i}+\sum_{i=1}^{n}\sum_{j=1}^{n}\beta_{\mathrm{ij}}x_{i}x_{j}|X=\left\{x_{1},x_{2},\ldots ,x_{n}\right\}$$

where $\beta_{0}$ is the independent coefficient, $\beta_{i}$ is the coefficient of the linear terms, and $\beta_{\mathrm{ij}}$ is the coefficient of the interactions. If $\beta_{\mathrm{ij}}=0\;\forall i,j$, we are in the presence of a multiple linear regression model, and if in addition n=1, Equation (1) becomes a simple linear equation.

#### 3.3.2. Decision Tree-Based Methods: Random Forest, GBM, and XGBoost

A decision tree model is a simple representation used for classifying examples or samples. It involves supervised machine learning, where the data are continuously divided according to specific rules applied to a particular parameter or a set of parameters [103,109]. In general, a decision tree consists of a root node, several interior nodes, and some terminal nodes. The root and internal nodes are connected by decision stages, while the terminal nodes represent the final classifications [110]. Tree models are simple, interpretable models. They have limited predictive power, but when several tree models are combined such as in clustered trees, random forest [24,111], boosting [25], gradient increasing, or extreme gradient boosting [100], they become a robust predictive model.

As an improvement to the decision tree algorithms explained above, assembler-type methods have emerged, formed by a group of predictive models called random forests, implying a significant improvement concerning individual decision trees. Random forest is a co-learning technique. It hybridizes the bagging algorithm [112] and the random subspace method and uses decision trees as the base classifier. Each tree is built from a starting sample of the original dataset. The important point is that the trees are not pruned after construction, which partially fits their data sample [113]. From random forests, progress is made toward boosting algorithms (boosting, gradient boosting, or XGBoost). Boosting is a highly effective and widely used machine learning method. Models are built sequentially, minimizing errors from previous models while increasing the influence of high-performance models. Boosting is a general set method that creates a robust classifier from several weak classifiers.

AdaBoost was the first genuinely successful boosting algorithm developed for binary classification. It is the best starting point from which to understand boosting. Modern reinforcement methods are based on AdaBoost. The AdaBoost algorithm begins by training a decision tree in which each observation is assigned a weight equal to $\omega_{i}=1/N,\;i=1,2,\ldots ,N$, increasing the weights of difficult-to-classify observations and lowering the weights of those that are easy to classify, cultivating the following trees with the data already weighted, and generating an aggregate model by incorporating more decision trees after calculating the classification errors. By repeating a specific number of iterations (M), the subsequent trees help to classify the observations that are not well classified by the previous trees. Therefore, the prediction of the final set model is the weighted sum of the predictions made by the previous tree models [114,115,116].

Gradient boosting is a generalization of the AdaBoost algorithm that allows any cost function to be used, as long as it is differentiable. The flexibility of this algorithm has made it applicable for boosting in respect of a multitude of problems, making it one of the most successful machine learning methods. Although there are several adaptations, the general idea of all of them is the same: train models sequentially so that each model adjusts the residuals (errors) of the previous models.

Gradient augmentation machines have shown considerable success in a wide range of practical applications, as they are highly customizable for particular application needs, such as learning different loss functions [101]. Gradient boosting also trains many models in a gradual, additive, and sequential way. The main difference when comparing with AdaBoost is how the two algorithms identify the weaknesses of the weak models. While the AdaBoost model identifies deficiencies using heavy data points, gradient augmentation does the same by using gradients in the loss function. This function indicates how good the model’s coefficients are in respect of fitting the underlying data. One of the major motivations for using gradient augmentation is that it enables a user-specified cost function to be optimized, rather than a loss function that generally offers less control and does not essentially correspond to real-world applications [117,118,119,120,121].

Finally, XGBoost is an end-to-end scalable tree augmentation machine learning method, a gradient augmentation algorithm optimized through parallel processing, tree pruning, missing value handling, and regularization techniques to avoid overfitting/biasing. XGBoost is an algorithm based on decision trees that have high accuracy and a low risk of overfitting, thanks to a boosting algorithm. XGBoost is based on gradient increase, which generates a robust classifier by iteratively updating the parameters of the previous classifier to decrease the gradient loss function, adding a regularization function to the loss function in the objective function. Another study of the mathematical formulation of the method can be found in Chen and Guestrin [100].

#### 3.3.3. Artificial Neural Networks

ANNs are supervised machine learning techniques that determine associations between a known set of observations (i.e., training points) and different environmental variables to classify new and unknown data [98]. The main advantages of ANNs are their ability to handle large datasets, approximate non-linear relationships, generalize complex systems from relatively imprecise information, and handle noise, overfitting, and outliers [122,123].

The most common artificial neural network structure type is the multilayer perceptron, which is composed of a structure with two or more hidden layers, where $X_{i}$ represents the inputs, $O_{i}$ the outputs, and f the activation function of each neuronal unit. The input layers depend on the information available to be classified (independent and/or operational variables). In contrast, the output layer has one or many nodes and is dependent on the number of response variables (dependent variables). The neurons in one layer are connected to those in the next layer by synapses. The value is different for each connection and is determined by the training process, the activation functions, and the initial values.

### 3.4. Validation Using Performance Measures

Once the models have been developed, they must be validated using different techniques. The measures of merit used in this study help determine the quality of the predictive models developed. The goodness-of-fit indicators are MAE/MAD, RMSE, and R^2^. These merit values are the mean absolute error (MAE) or mean absolute deviation (MAD), a measure of errors between paired observations expressing the same phenomenon (see Equation (2)); the root-mean-square deviation (RMSD) or root-mean-square error (RMSE), used to measure the differences between values (sample or population values) predicted by a model or an estimator and the values observed (see Equation (3)); and the coefficient of determination (R^2^), which is the proportion of the variation in the dependent variable that is predictable from the independent variable(s) (see Equation (4)).

(2) $$\mathrm{MAE}=\left(1/n\right)\sum_{i=1}^{n}\left|y_{i}-\hat{y}\right|$$

(3) $$\mathrm{RMSD}=\sqrt{\left(1/n\right)\sum_{i=1}^{n}{\left(y_{i}-\hat{y}\right)}^{2}}$$

(4) $$R^{2}=1-\sum_{i=1}^{n}{\left(y_{i}-\hat{y}\right)}^{2}/\sum_{i=1}^{n}{\left(y_{i}-\bar{y}\right)}^{2}$$

where $\hat{y}$ and $\bar{y}$ are the predicted and mean values of y. All indicators shown quantify how well a model fits a dataset.

## 4. Results and Discussions

### 4.1. Preliminary Analysis

**Figure 3:**
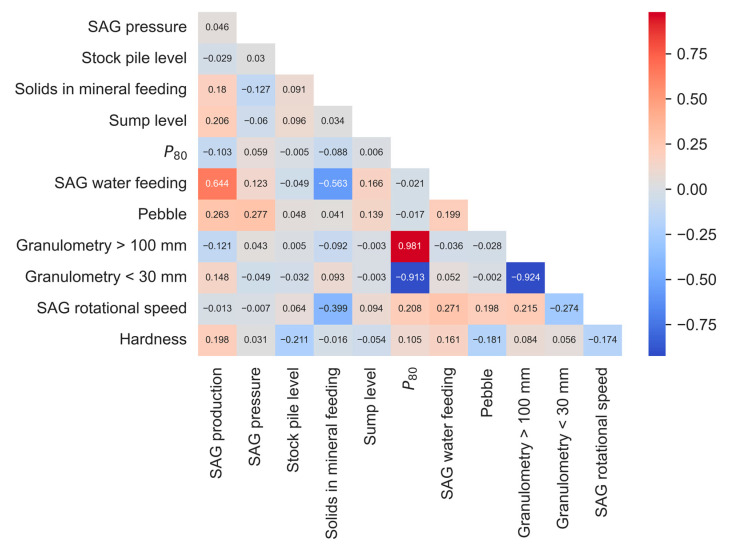
A Spearman correlation of the operational variables of an SAG grinding system.

To analyze the relationship between variables of interest (dependent and/or independent) considered in respect of sampling, a Spearman correlation analysis was developed. A Spearman correlation analysis relates the performance of a characteristic of interest to potential causal factors, explaining how each factor helps explain the response. The Spearman correlation study (see Figure 3) indicates a strong relationship between SAG production and SAG water feeding. In contrast, a moderate relationship is observed between SAG production and the following variables: sump level, pebble, and solids in mineral feeding. It should be noted that the non-existence of a monotonous relationship does not imply that these variables have no impact on the response, only that the impact (if any) is not monotonous (increasing or decreasing).

### 4.2. Validation of ML Algorithms

The adjustment of the SAG grinding system using machine learning algorithms can generate models of an SAG mill using algorithms such as multiple regression analysis, random forest, gradient reinforcement machines, and neural networks. Multiple linear regression models did not perform well, leaving evidence of the ineffectiveness of modeling the SAG mill dynamics using linear relationships.

Table 2 shows the goodness-of-fit indicators. The model based on a regression analysis does not allow modeling, examining, exploring spatial relationships, and explaining the factors behind the spatial patterns because the model does not present an adequate fit to the sampled dataset (R^2^ = 0.5514372). However, although the value of R^2^ indicates that the percentage of the variability of the response that the linear model explains is only approximately 55%, this percentage can be considered relatively good when considering the large amount of data and variables sampled that influence the dependent variable.

The gradient boosting machines (GBM) present a better fit than linear models, resulting in goodness-of-fit indicators of 75.46%, 77.18%, and 70.61%, for Ranger, XgBoost, and GBM, respectively. Extreme gradient boosting (XgBoost) is the method that presents the best performance out of the three methods indicated above (R^2^ = 77.18%). Such implementation of decision trees overcomes the defects of other machine learning methods based on decision trees (such as the Ranger method), which include the parallelization problem with respect to an increasing gradient and non-increasing in random forest. To overcome these defects, the algorithm employs two techniques: weighted quantile sketching and scatter-conscious divisions. The algorithm also uses second-order gradients (Newton boosting) to converge faster and advance regularization, improving the model’s generalizability and overcoming overfitting problems in terms of an increasing gradient [124].

**Figure 4:**
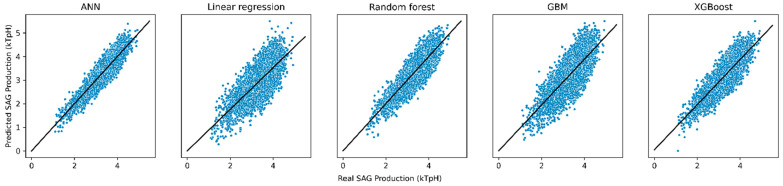
Scatterplots of real production versus predicted production of adjusted ML models.

The adjustment through the application of artificial neural networks, on the other hand, turned out to be the model with the best adjustment indicators (MAE, RMSE, and R^2^), where approximately 89% of the variability of the sample data is explained by the architecture of the adjusted artificial neural network (whose architecture corresponds to a multilayer perceptron). The low dispersion in the contrast of the fitted models versus the real production (kTpH) is shown in the scatter plots in Figure 4.

The digital model used to simulate the responses to variations for each of the strategies defined in the present case study is the model based on artificial neural networks, which presents the best goodness-of-fit indicators (see Table 2).

### 4.3. Application of the ML Model by Individual Strategies

#### 4.3.1. Mine-to-Mill

**Figure 5:**
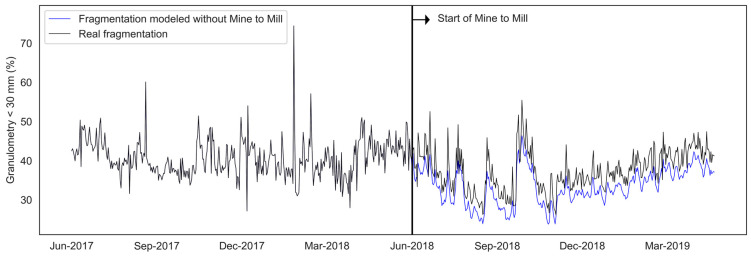
Daily average fragmentation of mineral feeding in an SAG mill.

**Figure 6:**
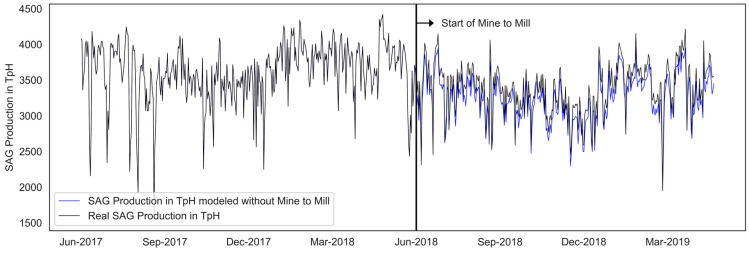
Average production of an SAG mill in tons per hour.

In the mine-to-mill strategy, the grinding process is simulated using the SAG mill digital model (ANN) to determine the level of fragmentation that tends to maximize the production of the SAG grinding process. The machine learning model was used to validate the impact of mine-to-mill on improving fragmentation (see Figure 5) and the increase in production in tons per hour (see Figure 6). The fragmentation after implementing the M2M strategy was compared with the fragmentation estimated without applying the method. The improvement was evaluated, quantifying the % increase in TpH as a function of the fragmentation improvement obtained with M2M.

**Figure 7:**
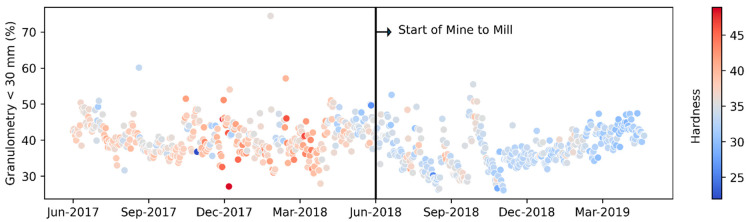
Variation of mineral hardness.

**Figure 8:**
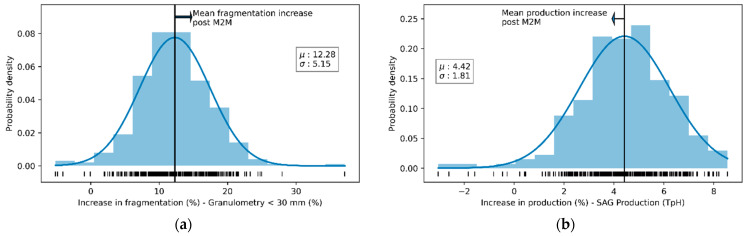
Distribution of the percentage of fragmentation improvements or the percentage of ore in feed < 30 mm (**a**) and the percentage increase in the SAG mill’s production in TpH (**b**).

The increase in granulometry (that is, for purposes of this work, the percentage of minerals less than 30 mm, a product of the application of the strategy) is approximately 12.28% (despite the increase in mineral hardness in the period, as is shown in Figure 7), with a standard deviation of 5.15 (see Figure 8a), while the increase in production is approximately 4.42% (see Figure 8b). It should be noted that the behavior of the percentage increase in granulometry and production tends to show normal behavior (see Figure 8).

**Figure 9:**
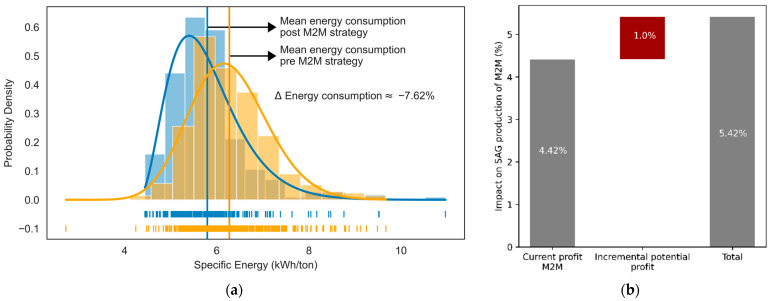
Specific energy consumption distributions (**a**) and the potential percentage impact on the production in TpH at the SAG mill (**b**).

In addition to quantifying the impact of M2M on production at +4.42% TpH, the model obtained −7.62% energy savings in the SAG mill (see Figure 9a). The ML model suggests that there would be the potential to increase TpH production by an additional 1% if the percentage of fine mineral increases (that is, fragmentation increases from 41% to 46%), prior to technical evaluation developed by the geology field. Then, the model suggests that the cost of the additional fragmentation delta derived from the comminution phases before SAG grinding could be quantified, which must be contrasted with the potential incremental profit of 1% in production using the M2M strategy (see Figure 9b).

#### 4.3.2. SAG Mill Parameter Control

The use of machine learning to improve the efficiency of the production process is focused on increasing the performance or production in TpH and reducing energy consumption. As the second optimization strategy for the SAG milling process, after identifying the variables of interest through the correlation study and the adjustment of the machine learning models presented in Section 3.3, the variable SAG rotational speed (RPM), solids in the feed (%), and SAG pressure (fill level) were fitted to evaluate TpH performance and energy consumption.

**Figure 10:**
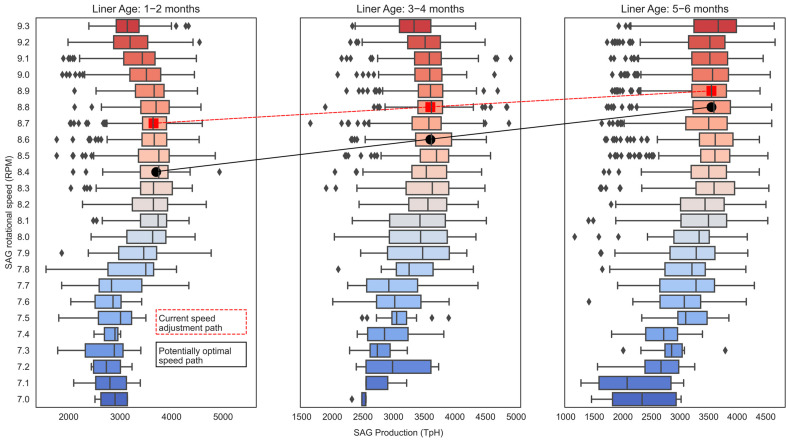
SAG production (TpH) versus SAG rotational speed (RPM) and liner age.

The analysis of the machine learning model for the study of production as a function of SAG rotational speed (RPM) and liner age (1–2, 3–4, 5–6 months) indicates that there is an optimal configuration speed that would allow an increasing TpH at lower SAG rotational speeds (RPM), regardless of the age of the liner, as shown in Figure 10. That is, there is the potential to increase production by decreasing the SAG rotational speed.

**Figure 11:**
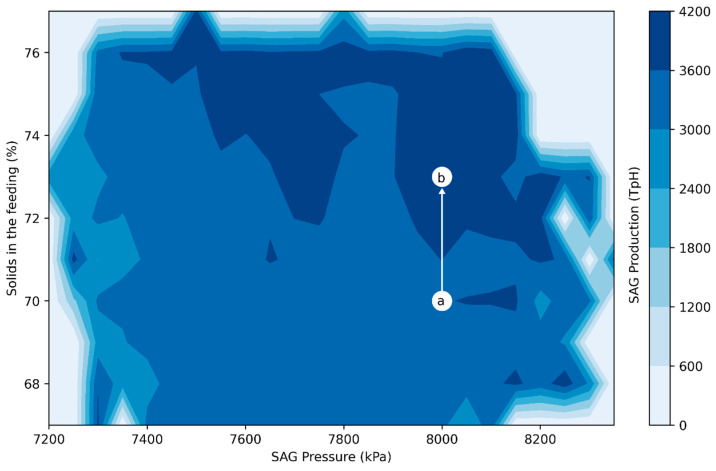
Optimum configuration of the percentage of solids and pressure in the SAG mill. The usual state of operation (a) and optimum state of operation (b).

After identifying the optimal linear level of the mill’s rotational speed and age, the optimal levels were used to determine the optimal configuration points for solid percentage and pressure (see Figure 11). Then, for a scenario of 1–2-month-old liners, a hardness > 32.5, and an optimal rotational speed of 8.4 RPM, the SAG mill is usually operated at a percentage of solids of 70% and a pressure of 8000 kPa (see Figure 11a). However, to improve the performance of the SAG production in TpH, the ML model suggests adjusting the percentage of solids to approximately 73% (see Figure 11b) while keeping the pressure constant. Additionally, the variation in the rate of solids and the decrease in the SAG rotational speed, in addition to increasing the production in TpH, decreases the probability of torque events (considering that mill pressure remains constant). It should be noted that the lower the torque, the lower the levels of energy consumption, and high torque levels prevent reaching optimal rotational speeds.

Then, optimal operating guidelines were constructed considering the operational parameters of the mill rotational speed, percentage of solids, and pressure for different hardness scenarios (<32.5, >32.5) and age of liners (1–2, 3–4, 5–6 months), as shown in Table 3.

**Figure 12:**
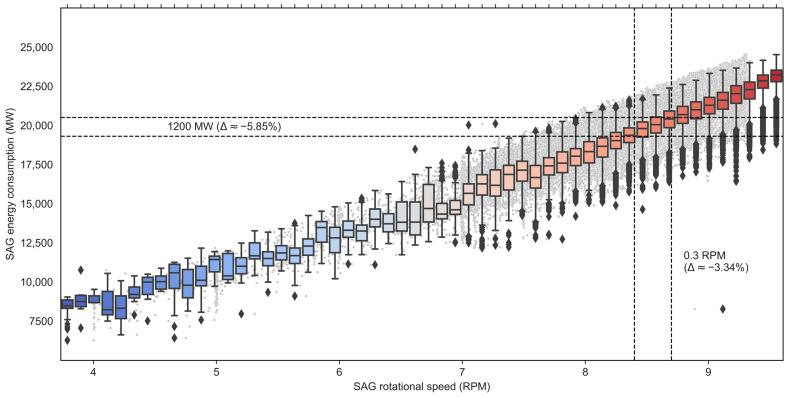
Correlation between SAG rotational speed (RPM) and SAG energy consumption (MW).

Finally, the machine learning model shows that to the extent that it is possible to operate with similar SAG production in TpH (slightly higher), reducing rotational speed allows a saving of 1.95% for every 0.1 RPM for the scenario where the hardness is greater than 32.5 and the liner age is 1–2 months (see Table 3). As shown in the correlation in the scatter plot and boxplots between SAG rotational speed and energy consumption (see Figure 12), the impact corresponds to approximately 5.85% of the cost of energy consumed by the SAG mill.

### 4.4. Discussion

Today’s world is economically volatile, and most mineral processing companies face challenges in creating sustainable plans and schedules over time, plans that directly affect the value chain. By combining machine learning techniques with data analysis, mining industries can improve the management of their production processes, developing models that allow simulation and optimizing operational indicators. In this way, recommendations can be obtained regarding the best way to operate while considering the domain of control variables.

Using adjusted machine learning models to represent the SAG grinding process, after identification and validation of the variables that have a direct impact on TpH performance, the variables that have a significant impact on the response were identified as liner age, hardness, granulometry, SAG rotational speed, percentage of solids, and the pressure level, while the response indicators were production in TpH and energy consumption. A summary of the different operational strategies (both the captured results and the incremental impact) is shown in Table 4, verifying both research hypotheses. It shows an increase in production in TpH as a result of an M2M strategy (H_1_) that produces a greater fragmentation of the mineral fed (which also impacts to a lesser degree on the energy consumption of the SAG mill), together with increases in production and decreases in energy consumption derived from optimum handling of mill control parameters, such as pressure and rotational speed, which has a limited impact on production growth but considerable savings in energy consumption (H_2_).

Finally, it highlights that machine learning or artificial intelligence algorithms are the keys to unlocking new findings in existing datasets (which are increasingly robust due to paradigms such as the Industrial Internet of Things, IIoT), such as those presented in the development of this work. IIoT technologies will allow mineral processing companies to collect, process, and generate intelligence from large amounts of data from sensors connected to the internet. These data could also be used to create digital twins, either from the plant as an aggregated process or for a particular thread (e.g., the SAG grinding process).

## 5. Conclusions and Future Works

### 5.1. Conclusions

Mineral processing modeling is often a difficult task due to the multiple variables involved and the complex dynamics that comprise the value chain. Despite its complexity and the application of many physical, mechanical, chemical, and other types of separation processes and operations, mineral processing can generally be divided into a reduction in the size of the feed material and a separation of useful minerals from the gangue [124]. As part of the physical treatment (a reduction in ROM), grinding is the most relevant sub-process since it is the one that consumes more resources as a result of the reduction in the particle size in large rotating equipment (such as SAG mills). In most mining operations today, comminution uses energy and inputs on an unprecedented scale, and the discovery of new mineral deposits in remote areas, with increasingly hard minerals and a demand for finely granulated downstream materials in mineral processing, have a significant influence on processing costs. Along with the above, there is a growing concern about the environmental impact of mining operations, which is why there is a focus on reducing energy consumption and reducing the carbon footprint of these industrial activities.

In the present work, models of an SAG mill were generated, adjusting different machine learning algorithms such as regression, decision trees, and artificial neural networks. The model that presented the best performance was based on artificial neural networks (R^2^ ≈ 0.89) and was used to study the research hypothesis of this work: the impact of fragmentation and the mill power strategy in TpH production. The conclusions reached are presented below:

- The application of the M2M strategy allowed for an increase in production of 4.41% in TpH and a potential increase of 1.0% (conditioned to a 3% increase in fragmentation); in addition, a decrease in energy consumption of 7.62% was observed (for a liner age of 1–2 months).
- The production modeling as a function of the mill power and the ages of the liners indicated that excess power is being applied. It is possible to increase the production in TpH by reducing the mill’s rotational speed, regardless of the age of the liners. The application of this strategy implies an increase of 0.76% in production and a decrease in energy consumption of 5.85% (only for a liner age of 1–2 months).

Algorithms based on ML are powerful tools for capturing the dynamics of complex systems, such as SAG grinding. It is possible to abstract the dynamics of operation (that is, the behavior of the explained variables based on the explanatory variables to simulate and/or optimize the model), find the conditions that optimize the output or, failing that, find a better configuration of variables that improves the process’s productive indicators.

### 5.2. Future Works

Despite the potential of the machine learning algorithms adjusted in the present work and the impact verified in operational conditions on the productive indicators, the dynamics of the grinding process involve variables that do not necessarily maintain deterministic behavior. The inclusion of stochastic variables or stochastic processes in the modeling, simulation, and optimization of the process will be of interest in future research [125]. The modeling of the dynamics of SAG grinding could also be applied in the implementation of circular economy strategies in the mineral processing industry with respect to the elevated energy consumption [126], aiming to make production more efficient and save energy costs, as observed in the development of the second hypothesis of this research.

Additionally, probability and sensitivity analysis (e.g., the Monte Carlo simulation method) could be applied to obtain the sensitivity of the explanatory variables versus the explained ones. These analyses are useful for generating confidence intervals for the estimated variables, while the sensitivity analysis would allow us to perform residual analysis, outlier detection, and assumption evaluation.

## Figures and Tables

**Table 1:** Operational variables of the SAG grinding process (V: Variable, R: Response; P: Parameter).

	Unit	Type	Lower Limit	Mean Value	Upper Limit
P_80_	mm	V	50	100	150
SAG water feeding	m^3^/h	V	750	1350	2000
SAG rotational speed	RPM	V	4	9	10
SAG pressure	kPa	R/P	7300	7700	8100
Stockpile level	m	V	5	25	35
Sump level	m	R/P	60	90	100
Hardness	dimensionless	P	20	35	50
Solids in the feeding	%	V	55	70	80
Pebble	TpH	R/P	0	400	900
Granulometry > 100 mm	%	V	5	20	40
Granulometry < 30 mm	%	V	25	40	75
Liner age (LA)	months	P	1–2	3–4	5–6

**Table 2:** Goodness-of-fit statistics for statistical and machine learning techniques.

Model	RMSE	R^2^	MAE/MAD	RMSESD	R^2^SD	MAESD
MR	411.1305	0.5514372	320.3928	11.53500	0.02511	8.62293
Random Forest	330.7013	0.7546393	249.8718	10.12509	0.02463	8.22492
XGBoost	319.9871	0.7717928	243.1334	8.863804	0.01905	6.69247
GBM	349.1443	0.7060547	269.4338	6.862753	0.03074	6.64043
ANN	284.6292	0.8852025	203.9580	5.232471	0.01435	5.23569

**Table 3:** Optimal operating guidelines for the SAG mill.

Hardness	Liner Age	Rotational Speed (RPM)		Solids in the Feeding (%)		Pressure (kPa)	
		Actual	Optimum	Actual	Optimum	Actual	Optimum
>32.5	1–2	8.7	8.4	72	75	7960	8000
	3–4	8.7	8.5	72	75	7813	7900
	5–6	8.8	8.7	72	75	7556	7700
<32.5	1–2	8.6	8.5	72	72	7934	8000
	3–4	8.9	8.7	72	75	7744	7700
	5–6	8.7	8.7	72	75	7540	7600

**Table 4:** The impact of the ML model on the SAG grinding system’s operational strategies (LA: liner age).

Strategy	Impact on TpH		Impact on Energy
	Captured	Incremental	
Mine-to-mill: Identification of the current impact of granulometry and its additional potential	+4.41%	+1.0%	−7.62%
SAG parameters: Optimal operation for LA and hardness scenarios		+0.76%	−5.85% (LA: 1–2)

## Data Availability

The data that support the findings of this study are available from the corresponding author, M.S., upon reasonable request.
